# Pulp stem cells with hepatocyte growth factor overexpression exhibit dual effects in rheumatoid arthritis

**DOI:** 10.1186/s13287-020-01747-y

**Published:** 2020-06-10

**Authors:** Xiwen Dong, Fanxuan Kong, Chao Liu, Shiyun Dai, Yuning Zhang, Fengjun Xiao, Huan Zhang, Chu-Tse Wu, Hua Wang

**Affiliations:** 1 Department of Experimental Hematology, Beijing Institute of Radiation Medicine, 27 Taiping Road, Beijing, 100850 People’s Republic of China; 2 Beijing Key Laboratory for Radiobiology, Beijing Institute of Radiation Medicine, Beijing, 100850 People’s Republic of China; 3 Binzhou Medical University, Yantai, 264003 Shandong People’s Republic of China; 4 The Fifth Department of Chemotherapy, Affiliated Tumor Hospital of Guangxi Medical University, Nanning, 530021 People’s Republic of China

**Keywords:** Dental pulp stem cells (DPSCs), Hepatocyte growth factor (HGF), Rheumatoid arthritis (RA), Cell therapy

## Abstract

**Background:**

To investigate the therapeutic effect of human dental pulp stem cells (DPSCs) transfected with adenovirus expressing hepatocyte growth factor (HGF) in a mouse model of collagen-induced arthritis (CIA).

**Methods:**

DPSCs were modified with Ad-HGF to produce HGF-overexpressing DPSCs, DPSCs-HGF. In experimental mouse CIA model, DPSCs-HGF and DPSCs-Null (modified with Ad-Null) were engrafted via intravenously after disease onset, which was determined by the presence of joint swelling. The therapeutic effects on joints were evaluated at 49 days after collagen injection by histopathological analysis and microcomputed tomography imaging. The inflammatory cytokines were analyzed both in sera and joints via MILLIPLEX kit and immunohistochemical staining, respectively, and the regulatory T cells (Tregs) were analyzed in peripheral blood by using flow cytometry. Furthermore, primary fibroblast-like synoviocytes were isolated, colony formation analysis and FACS were performed to evaluate the effect of HGF on the proliferation and cell cycle of FLSs. Western blot assay was carried out to clarify the signal pathway of HGF-cMet.

**Results:**

We found that without HGF modification, DPSC transfusion was helpful in controlling autoimmune status, local synovitis, and bone erosion after intravenous administration. However, HGF-modified DPSCs have dual role in rheumatoid arthritis (RA). In the early phase, HGF overexpression inhibited RA progression by its immunosuppressive effects, while in the late phase, HGF promoted synovitis by activating fibroblast-like synoviocytes to produce pathogenic IL-6, accelerating cell proliferation and inducing apoptosis resistance via phosphorylating the c-Met/Akt pathway. The overall effect of HGF modification attenuated the therapeutic effect of DPSCs.

**Conclusions:**

Our study provides a comprehensive evaluation of the therapeutic effect of DPSCs in the mouse model and a primary answer to the divergence of whether HGF is harmful or helpful in RA.

## Introduction

RA is one of the most prevalent chronic autoimmune diseases, with an incidence of 0.5–1% in adults worldwide [1]. RA patients have both articular symptoms (pain, stiffness, swelling, etc.) and extra-articular manifestations (pleurisy, hydrothorax, anemia, etc.) as a result of autoimmune activation, prolonged inflammation, cartilage deterioration, and bone destruction [2]. Innate and adaptive immune cells as well as synovial cells participate in RA pathogenesis [3, 4]. Innate immune cells, such as macrophages and dendritic cells, facilitate the breakdown of immune tolerance. After that, adaptive immune cells (such as T or B lymphocytes) are activated, causing transformation of synovial cells to a more aggressive phenotype, which contributes to joint damage, and enhancing osteoclast activity, which induces osteolysis [3].

Disease-modifying anti-rheumatic drugs (DMARDs) and nonsteroidal anti-inflammatory drugs (NSAIDs) are the two major first-line drugs in RA treatment [1]. In terms of a treatment-to-target strategy, the prospects for most patients are now favorable, yet approximately 30% still do not respond to current drugs. Even when clinical remission is achieved, cartilage and bone erosion may stealthily persist resulting from ongoing autoimmune reaction [5]. Therefore, a promising strategy is to restore prolonged immune tolerance and to protect against local joint damage [6].

Recent evidence demonstrated that mesenchymal stem cells (MSCs) can reshape the immune response by altering both innate and adaptive immunity through direct cell-cell contact or secretion of modulatory factors. The immunomodulatory effects of MSCs have been extensively studied in MSCs from diverse tissue sources (such as bone marrow, adipose tissue, and dental pulp) [7]. As an important type of MSC, DPSCs are the first human dental stem cells extracted from dental pulp, which possess the capacity of immunomodulation, multilineage differentiation, and self-renewal [8]. DPSCs have been reported to possess stronger immune-regulatory effect than that of BMSCs [9, 10]. Conditioned medium from human deciduous DPSCs was reported to be helpful in anti-collagen type II antibody-induced arthritis. Moreover, in terms of their strong osteogenic capacity, DPSCs are an ideal cell source for bone regeneration. For instance, Kong et al. reported that DPSCs could prevent bone loss in ovariectomy-induced osteoporosis [11]. However, DPSCs’ prevention of bone erosion in RA needs to be studied.

HGF is a multifunctional growth factor that plays a vital role in embryogenesis and organ development [12]. It could suppress acute and chronic inflammation after binding to its receptor c-Met. HGF/c-Met can modulate both innate and adaptive immune responses by stimulating anti-inflammatory cytokine secretion and inducing tolerogenic dendritic cells, respectively [13]. It has been reported that HGF delivery by hydrogel could suppress antigen-induced T cell priming and enhance the Th2-type immune response to inhibit the development of CIA [14]. However, whether HGF modification could synergy DPSC therapeutic effect remains to be elucidated.

In the present study, CIA mice were used and treated with phosphate buffer saline (PBS), DPSCs transfected with control adenovirus vector (DPSCs-Null), or DPSCs transfected with HGF adenovirus vector (DPSCs-HGF). The curative effect of each treatment was measured by clinical and histological scores, flow cytometer analysis, and micro-CT imaging. Furthermore, to study the underlying mechanism, FLSs from RA patients were isolated and cultured in different conditions. For the first time, we demonstrated the therapeutic effect of DPSC transfusion in RA and revealed the dual effects of HGF in RA pathogenesis, all of which represent new insights as well as a novel strategy for RA treatment.

## Materials and methods

### Human DPSC preparation

DPSCs were isolated and cultured as previously described [15, 16]. All experiments follow the Declaration of Helsinki. Briefly, after patients (male, 19–29 years old) signed the informed consent, their impacted third molars were completely removed at the Dental Clinic of Beijing Stomatological Hospital following standard procedures. The experiments were approved by the Research Ethics Committee of Capital Medical University, China.

The DPSCs were isolated and cultured in a GMP-compliant facility following ISO 8 clean room standards. Before digestion in collagenase, the pulp tissue was revealed and separated from the crown and root. Animal origin-free collagenase (Worthington Biochemical Corporation, Lakewood, NJ, USA) was used to digest the pulp tissue. Xenobiotic-free cell culture reagents were used for cell culture. Detailed information on the cell culture and characterization of DPSCs was well documented in previous articles [15, 17]. To maintain stable and reliable results, the DPSCs used in the experiments were at passage 3–4 (approximately 15–20 divisions of the primary DPSCs).

### Adenoviral vectors and gene transfection

Two kinds of adenovirus vectors (AdVs) were used in the study: Ad-HGF, a replication-defective adenovirus expressing human HGF, and Ad-Null, a replication-defective adenovirus not carrying exogenous genes. Both vectors were constructed with the AdEasy system (Stratagene, La Jolla, CA, USA) as previously described [18]. DPSCs were infected at a multiplicity of infection (MOI) of 150. After infection with Ad-HGF or Ad-Null for 2 days, the cells were collected and transfused into CIA mice through the caudal vein. Successful overexpression of HGF was verified by western blotting (Supplementary Fig. 1A).

### Induction of the CIA model

DBA/1 mice were purchased from Beijing Vital River Laboratory Animal Technology Co., Ltd. (Beijing, China) and were housed in the SPF facility within a stable environment (temperature 20–24 °C, humidity 45–65%, and 12 h light-dark cycle). The CIA model was induced by intradermal injection of complete Freund’s adjuvant (CFA, Chondrex, WA, USA) and bovine type II collagen (CII, Chondrex, WA, USA) emulsion in the area 1.5 cm distal to the base of the tail. Fourteen days after the first immunization, incomplete Freund’s adjuvant (IFA, Chondrex, WA, USA) and the CII emulsion were injected (proximal to the primary injection site) to boost the immune response [19, 20].

After the second immunization, the severity scores were evaluated for each mice (*n* = 10) as previously described twice every week [19]. The severity score was evaluated according to the following scale: 0 = no swelling or erythema, 1 = erythema and/or slight swelling restricted to the tarsals or ankle joint, 2 = erythema and/or slight swelling from the ankle to the tarsals, 3 = erythema and pronounced swelling extending from the ankle to the metatarsal joints, and 4 = erythema and severe swelling encompassing the ankle, foot, and digits or ankylosis of the limb [19]. The severity score is the average score of each arthritic mouse.

After disease onset, which was determined by the presence of joint swelling, cell treatment was carried out. Mice were intravenously injected with PBS, 1 × 10^6^ DPSCs-Null cells or 1 × 10^6^ DPSCs-HGF cells.

### Histological and immunohistological evaluation of mice joints

The mouse joints were fixed in paraformaldehyde and decalcified with EDTA (Servicebio Technology, Wuhan, Hubei). Tissues were paraffin-embedded, sliced, and stained with hematoxylin and eosin for morphological quantification evaluation. To evaluate the immunological and inflammatory changes of synovitis after treatment, the histopathological synovitis score was reviewed using CaseViewer software (3D HISTECH, Hungary) following guidance proposed by Krenn [21]. The score equaled the sum of three key aspects of synovitis, namely, synovial surface width, stroma density, and inflammatory infiltration. Each aspect contained 4 semiquantitative levels (normal 0, mild 1, moderate 2, and severe 3). Histological assessments were made under double-blind conditions and repeated at least twice.

Immunohistochemical staining was carried out using a streptavidin–peroxidase kit (Zymed, CA, USA) in accordance with the manufacturer’s protocols. After epitope retrieval and blockage of endogenous peroxidases, tissues were incubated with antibodies against IL-6 (1:200, Proteintech, Hubei, China) overnight at 4 °C. Irrelevant mouse/rabbit IgG (Abcam, CA, USA) was used as an isotype control. Sections were then successively incubated with biotin-labeled goat anti-mouse/rabbit IgG, horseradish peroxidase-labeled streptavidin, and diaminobenzidine. Finally, hematoxylin was used to stain the nuclei.

Each sample was analyzed independently. Only clear staining of cell cytoplasm (IL-6) was considered positive. Histochemistry scores (H scores) were calculated to check the expression of target molecules [22]. In short, H scores resulted from semiquantitative evaluation of the dominant intensity pattern of staining (1, negative or trace; 2, weak; 3, moderate; and 4, intense) multiplied by the percentage of positive cells per slide (0 to 100%).

### Microcomputed tomography (micro-CT) imaging

The right hind limbs of the mice were collected and analyzed by a high-resolution micro-CT imaging system (Inveon MM CT; Siemens, Munich, Germany) to assess bone volume, set to a 9.08-μm effective pixel size in the mouse model. The bone volume of the hind paw was assessed in a 0.5-mm region 0.5 mm below the distal growth plate of the femur, and the cortical bone was assessed in a 1-mm region 5 mm below the distal growth plate of the femur. The scanning, analysis, and 3D rebuild software used were Inveon Acquisition Workplace (Siemens), Inveon Research Workplace (Siemens), and COBRA (Exxim, Pleasanton, CA), respectively.

### Primary human FLS preparation

The study has been approved by the Ethics Committee of Chinese People’s Liberation Army General Hospital, China. Informed consent has been obtained from patients before FLSs isolated. Human FLSs were prepared according to previously reported procedures [23]. In brief, synovial tissues from patients were harvested after orthopedic operations and were separated from adipose tissues prior to enzymatic digestion with collagenase II (4 mg/ml; Sigma, MO, USA) for 2 h at 37 °C. After washing twice with PBS, the cell pellet was resuspended in DMEM/F12 (Gibco, NY, USA) containing 10% fetal bovine serum (FBS, Gemini, PA, USA) and 1% penicillin/streptomycin. FLSs were then cultured at 37 °C in a humidified atmosphere of 5% CO_2_. Cells used for the experiments were at passage 3–4, which were more purified and more biologically stable than cells above passage 5. Human recombinant HGF and TNF-α were purchased from PeproTech (NJ, USA). Inhibitors, such as JNJ-38877605 (c-Met pathway), LY294002(Akt pathway), and YM-155(Survivin expression), were purchased from MedChem Express (NJ, USA).

To detect the stimulation effect of HGF, FLSs were cultivated in culture media containing DPSC culture supernatant (1:3 v/v), culture media containing HGF (100 ng/ml), or culture media containing DPSC culture supernatant plus HGF (1:3 v/v). To inhibit certain pathways, FLSs were treated with 10 μM LY294002, 200 nM JNJ-38877605, or 100 nM YM-155 for 12 h. FLSs were then stimulated with 200 ng/ml HGF for 12 h before detection.

### Colony formation analysis

FLSs were seeded in 6-well plates at a density of 200 cells per well. To detect the stimulation effect of HGF, FLSs were cultivated in culture media containing DPSC culture supernatant (1:3 v/v), culture media containing HGF (100 ng/ml), or culture media containing DPSC culture supernatant plus HGF (1:3 v/v). To inhibit certain pathways, FLSs were treated with 5 μM LY294002, 100 nM JNJ-38877605, or 50 nM YM-155. After culturing for 14 days, colonies were fixed and stained with crystal violet solution.

### Western blot assay

After treatment, FLSs in 6-well plates were washed with cold PBS three times and lysed in RIPA lysis buffer (Beyotime, Shanghai, China) supplemented with premixed protease and phosphatase inhibitors (Beyotime, Shanghai, China) for 15 min on ice. Supernatant was collected by centrifugation of 13,000*g* at 4 °C for 20 min. Before electrophoresis, total proteins were quantified by bicinchoninic acid protein assay (BCA, Thermo scientific, Rockford, USA) and boiled for 8 min in loading buffer. Under constant voltage, proteins were resolved in 10% or 12.5% polyacrylamide gels before being transferred to PVDF membranes. Then, the membranes were blocked in 3% BSA and incubated with the corresponding primary antibodies overnight at 4 °C. The membranes were then washed in Tris-buffered saline containing Tween-20 (TBST) 3 times (5 min each) and incubated in secondary HRP-conjugated secondary antibodies for 1 h at RT. Signals were visualized by ECL western blotting substrate (Solarbio Life Science, Beijing, China) and detected by using a Tanon imaging system (Tanon, Shanghai, Beijing). Each experiment was carried out at least twice.

Primary antibodies used in immunoblot and immunoprecipitation assays were anti-HGF (1:1000, Proteintech, Hubei, China), anti-phospho-Met (1:1000, Cell Signaling Technology, MA, USA), anti-c-Met (1:1000, Proteintech, Hubei, China), anti-phospho-Akt (Ser473) (1:1000, Cell Signaling Technology, MA, USA), anti-Akt (1:1000, Cell Signaling Technology, MA, USA), anti-survivin (1:800, Proteintech, Hubei, China), anti-cleaved-Caspase-3 (1:1000, Proteintech, Hubei, China), anti-Caspase-3 (1:1000, Proteintech, Hubei, China), anti-GAPDH (1:1000, Cell Signaling Technology, MA, USA), anti-CDK1 (1:1000, Proteintech, Hubei, China), and anti-Cyclin B1 (1:1000, Proteintech, Hubei, China).

The secondary antibodies used in the immunoblot assay were goat anti-rabbit IgG-HRP (1:3000, Beyotime, Shanghai, China) and goat anti-mouse IgG-HRP (1:3000, Beyotime, Shanghai, China).

### Cytokine analysis

After collection, mouse serum was kept at − 80 °C. IL-6 and TNF-α levels were measured using a commercially available MILLIPLEX kit (ProcartaPlex Mouse Th1/Th2 & Chemokine Panel 1). The data were collected by MAGPI and analyzed with Milliplex Analyst (Millipore) software. Interleukin (IL)-6 levels in cell culture media were measured by enzyme-linked immunosorbent assay with a human IL-6 ELISA kit (DAKEWE, Guangdong, China) according to the manufacturer’s instructions. The absorbance at 450 nm was read by an ELISA plate reader.

### Flow cytometry

Peripheral blood was taking from canthus vein and kept in EP tubes with heparin sodium on ice before staining. To analyze the ratio of Treg cells in the blood, we used Mouse Regulatory T Cell Staining Kit 1# (eBioscience) according to manufacturer’s protocol. Briefly, after fixation/permeabilization and washing, cells were blocked with anti-mouse CD16/CD32 for 15 min, followed by staining for PE-FoxP3, FITC-CD4, and APC-CD25 for 30 min. After washing twice, samples were collected and analyzed by FACSCalibur (Becton Dickinson Corporation, USA).

### Cell cycle analysis

The cell cycle was analyzed by propidium iodide (PI) staining. Briefly, after collecting cells from 6-well plates, cells were fixed with 70% ethanol for 24 h. Then, the cells were washed twice in PBS. Cells were stained with 50 μM PI containing 5 μg/ml RNase A for 0.5 h and analyzed by flow cytometry (FCM) using a Calibur (Beckman Coulter, CA, USA). Cell cycle results were analyzed by Modfit Software.

### Statistical analysis

Statistical analysis was carried out using SPSS. Comparisons between groups were measured by ANOVA and post hoc analysis. In the graphs, the data are expressed as the mean ± SD. *p* values < 0.05 were considered statistically significant.

## Results

### DPSCs have therapeutic effects in controlling CIA joint damage

**Fig. 1:**
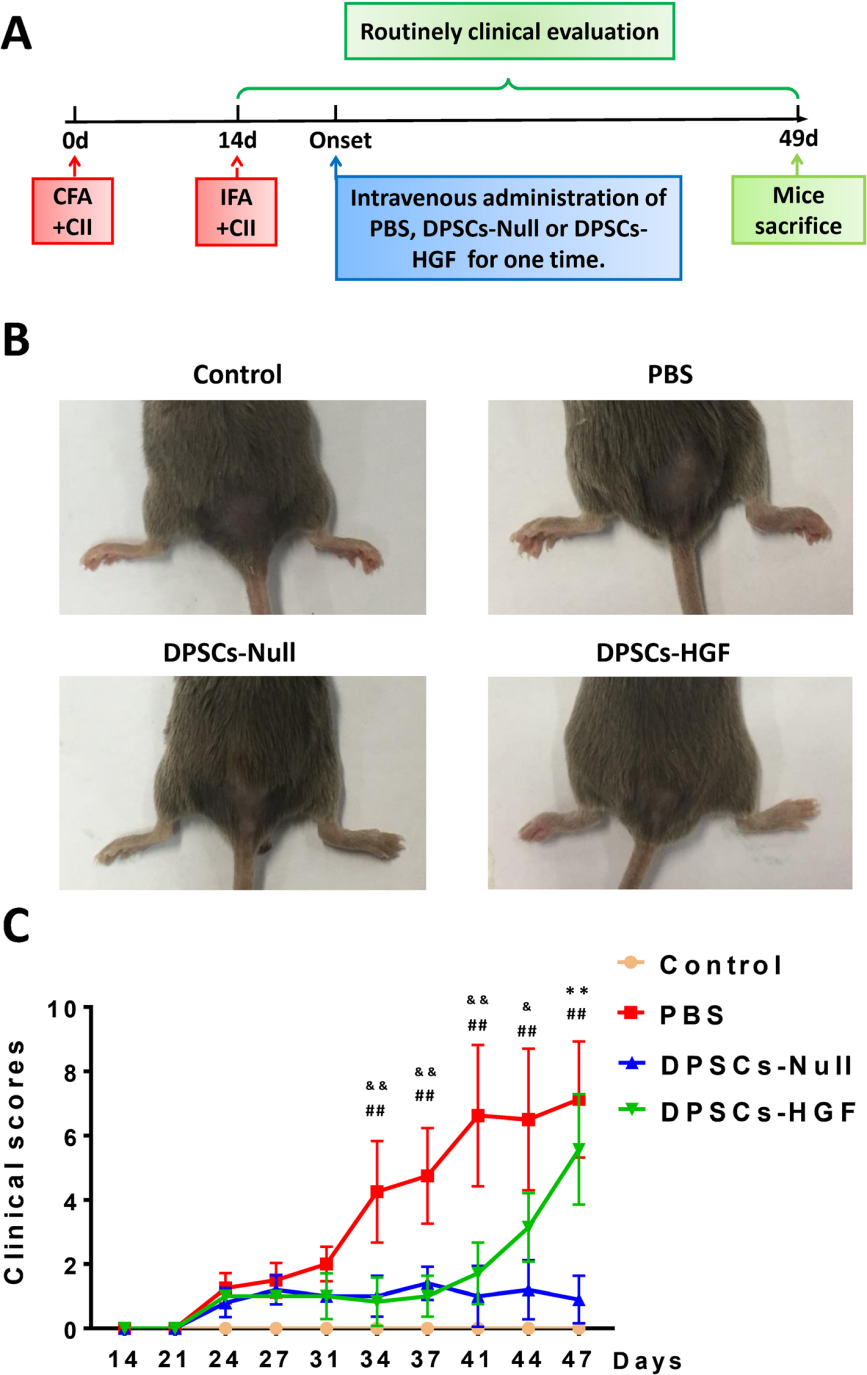
Clinical manifestation of CIA in mice after different treatments. **a** The flow diagram shows the study design of immunization and the following analysis. **b** The photographs represent the typical inflamed joints. Severe arthritis presents as joint rashes, swelling, and stiffness. The experiments have repeated once. **c** Clinical evaluation is presented in the statistical charts. The line graph shows the clinical scores of inflamed mice. The clinical score of each mouse was the sum of the arthritic limbs. For each limb, the quantification was conducted according to the criteria using a scale of 0–4, with 4 being the most severe inflammation. The mean severity score is the average score of each arthritic mouse. Incarnadine line with dots represents normal control group, red line with squares represents the PBS group, blue line with triangles represents the DPSCs-Null treatment group, and green line with triangles represents the DPSCs-HGF treatment group (each group contains 10 mice. # represents *p* < 0.05, ## represent *p* < 0.01 (PBS group vs. DPSCs-Null group); & represents *p* < 0.05, && represent *p* < 0.01 (PBS group vs. DPSCs-HGF group); ** represent *p* < 0.01 (DPSCs-Null group vs. DPSCs-HGF group)

A flow diagram (Fig. 1a) depicts the design of the experiment. The CIA model was established by two rounds of immunization on days 0 and 14 with CFA + CII and IFA + CII, respectively. Clinical evaluation of arthritis score was then performed routinely twice every week. After joint rashes or mild swelling appeared, PBS, DPSCs-Null, or DPSCs-HGF was intravenously administered to specific groups of mice. The inflammation status of each mouse was evaluated by flow cytometry and cytokine analysis. When mice were sacrificed on day 49, joint samples were collected for further analysis.

The photographs in Fig. 1b represent typical changes after receiving specific treatments. The DPSCs-Null group had milder joint swelling and fewer rashes on the joint surface than the other groups, including the PBS group. In addition, the arthritis score for each affected joint (Fig. 1c) in the DPSCs-Null group (blue line with triangles) was much lower than that of the PBS group (red line with dots) and was perfectly controlled at a low level since day 24, indicating that DPSCs-Null treatment could alleviate symptoms in CIA mice with mild joint swelling.. These results demonstrated that DPSCs were capable of suppressing CIA deterioration and may be beneficial to RA joint protection.

**Fig. 2:**
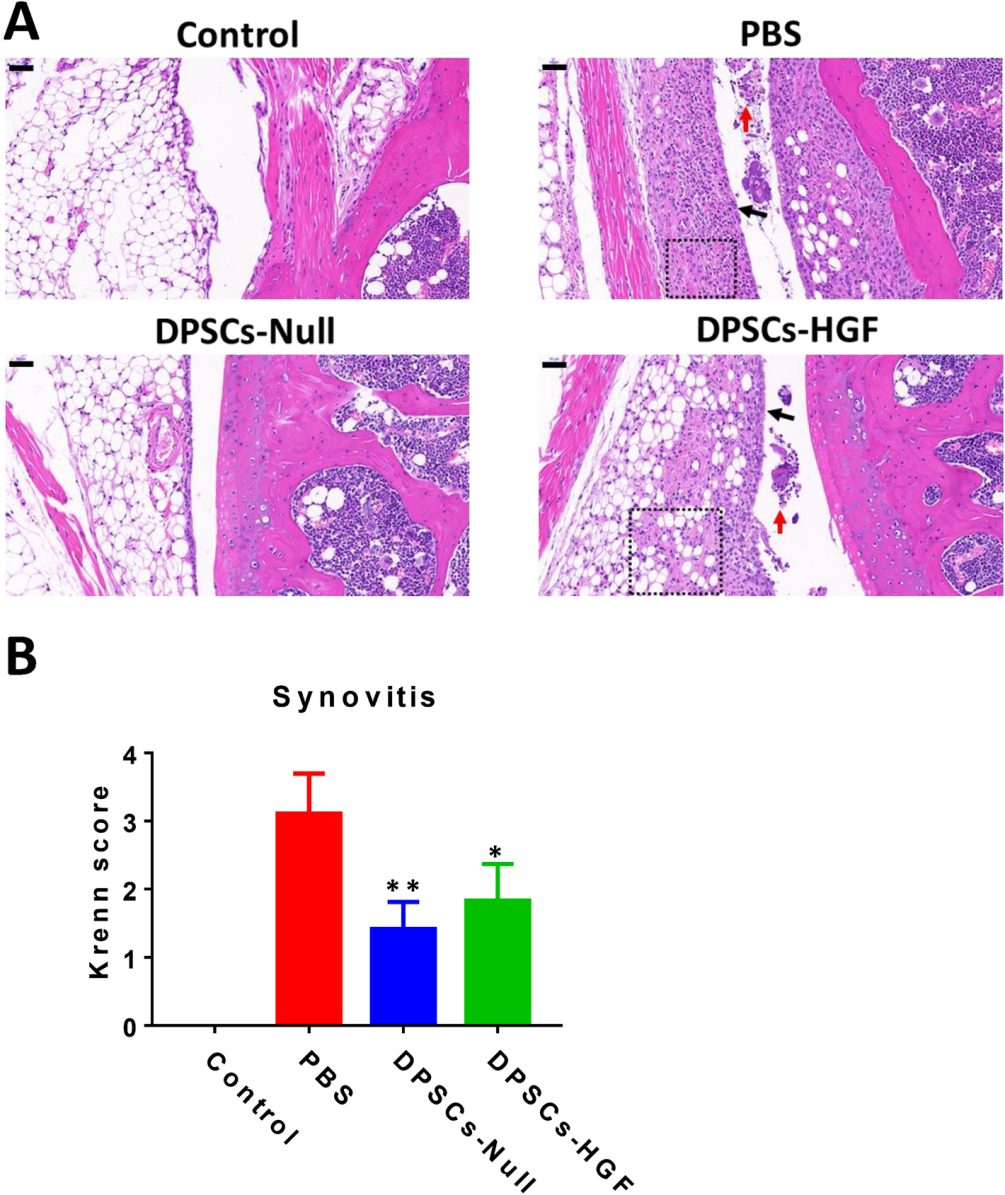
Synovitis evaluation in CIA mice after different treatments. **a** Examples of H&E staining results representing local joint damage in each mouse. Black dotted box: increasing density of resident cells; black arrow: inflammatory cell infiltration; red arrow: thicker synovial lining cell layers. Bar = 50 μm. The assessment was performed after the mice were sacrificed on day 49. **b** Histopathological assessment of synovitis in different groups. The synovitis score is the sum of the scores of the following aspects: the enlargement of the synovial cell layer, the density of resident cells, and the infiltration of inflammatory cells, with each aspect ranging from 0 to 3 points. ** represent *p* < 0.01 (DPSCs-Null group vs. PBS group) and * represents *p* < 0.05 (DPSCs-HGF group vs. PBS group)

To confirm the joint protection effect of DPSCs, pathological analysis (H&E staining) was carried out to reveal the pathologic structure (Fig. 2a). Compared with the levels in the DPSC treatment group, the PBS group showed a higher density of resident cells (black dotted box), more inflammatory cell infiltration (black arrow), thicker synovial lining cell layers (red arrow), and more intact structure. The Kreen score [21] for synovitis was calculated (Fig. 2b), indicating that DPSC transplantation was beneficial to CIA treatment.

### Overexpression of HGF may not be helpful for long-term CIA treatment

Intriguingly, overexpression of HGF did not provide satisfactory therapeutic effects as expected. During the early period (less than 37 days), the DPSCs-HGF treatment (green line with solid triangles) maintained the clinical score of each affected limb at a very low level (Fig. 1c). However, after day 41, both the arthritis score and incidence of the DPSCs-HGF treatment group soared intensively, while those of the DPSCs-Null treatment group began to decrease.

Similar to the clinical evaluation, the joint structures of the DPSCs-HGF treatment group were not much improved compared to those of the DPSCs-Null treatment, with more pannus formation and infiltration of inflammatory cells (Fig. 2a). The Kreen score (Fig. 2b) demonstrated that HGF overexpression interfered with the therapeutic effect of DPSCs.

### DPSCs could modulate the immunologic properties of CIA mice

**Fig. 3:**
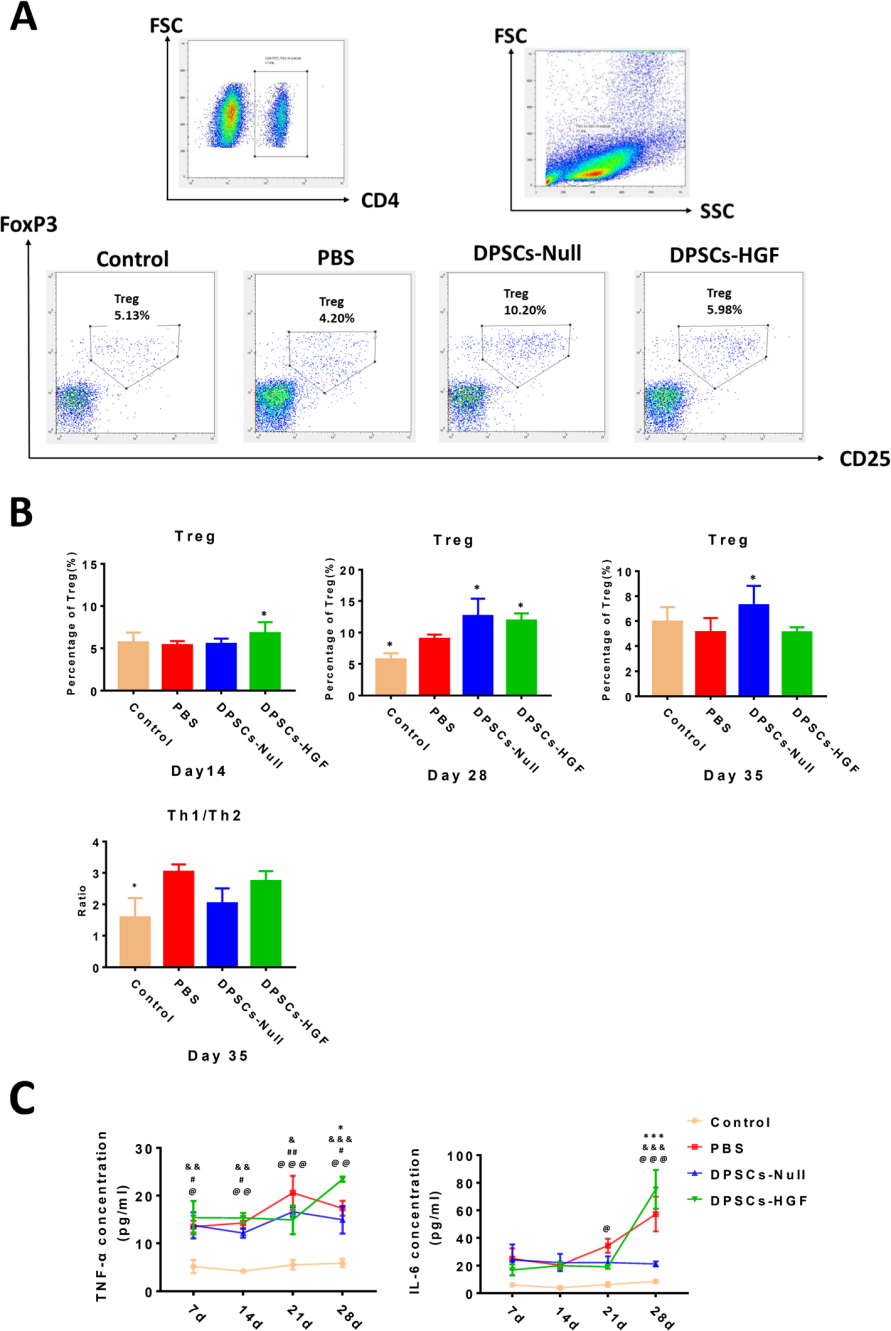
Immunologic properties of CIA mice after different treatments. **a** Examples of gating for peripheral blood Treg cells in different groups on day 28. **b** Statistic charts depict the Treg cell ratio in CD4^+^ T cells and the Th1/Th2 cell ratio. * represents *p* < 0.05 (other groups vs. PBS treatment group). *n* = 5. **c** Statistical analysis of serum IL-6 and TNF-α (incarnadine line with dots represents the control group, red line with squares represents the PBS group, blue line with triangles represents the DPSCs-Null treatment group, and green line with triangles represents the DPSCs-HGF treatment group, *n* = 5). @ represents *p* < 0.05, @@ represent *p* < 0.01, @@@ represent *p* < 0.001 (control group vs. PBS group); # represents *p* < 0.05, ## represent *p* < 0.01 (control group vs. DPSCs-Null group); & represents *p* < 0.05, && represent *p* < 0.01, &&& represent *p* < 0.001 (control group vs. DPSCs-HGF group); * represents *p* < 0.05, *** represent *p* < 0.001 (DPSCs-Null group vs. DPSCs-HGF group)

Since Treg cells are the major protective T cells in RA, we evaluated the presence of Treg cells in different groups (Fig. 3a). On day 14, the DPSCs-HGF treatment group possessed the highest level of Treg cells, suggesting that the immunosuppressive effect of the DPSCs-HGF group occurred earlier than that of the DPS-Null group (Fig. 3b, left). Then, during the next 14 days, Treg cells in the DPSCs-Null group began to increase sharply and reached a peak on day 28 (Fig. 3b, middle). After that, Tregs in both the DPSCs-Null and DPSCs-HGF groups began to decrease, yet Treg cells in the DPSCs-Null group lasted longer than those in the other groups (Fig. 3b, right). Besides, Th1/Th2 ratio were also elevated in PBS and DPSCs-HGF treated group on day 35 (Fig. 3b, down).

In addition, as the two major pathogenic factors in RA, TNF-α and IL-6 levels in serum were measured (Fig. 3c). Both TNF-α and IL-6 increased in model groups compared with normal control group, indicating the pro-inflammation change after immunization. Besides, unlike TNF-α’s change pattern, IL-6 seems to be more relevant to clinical changes. DPSCs-Null treatment holds IL-6 in a lower level (at around 20 pg/ml), yet DPSCs-HGF treatment pulled it up nearly 4 times on day 28. This indicated that IL-6 could be the reason for deterioration in DPSCs-HGF treatment group.

### Overexpression of HGF attenuates the protective effect of DPSCs in cartilage damage

**Fig. 4:**
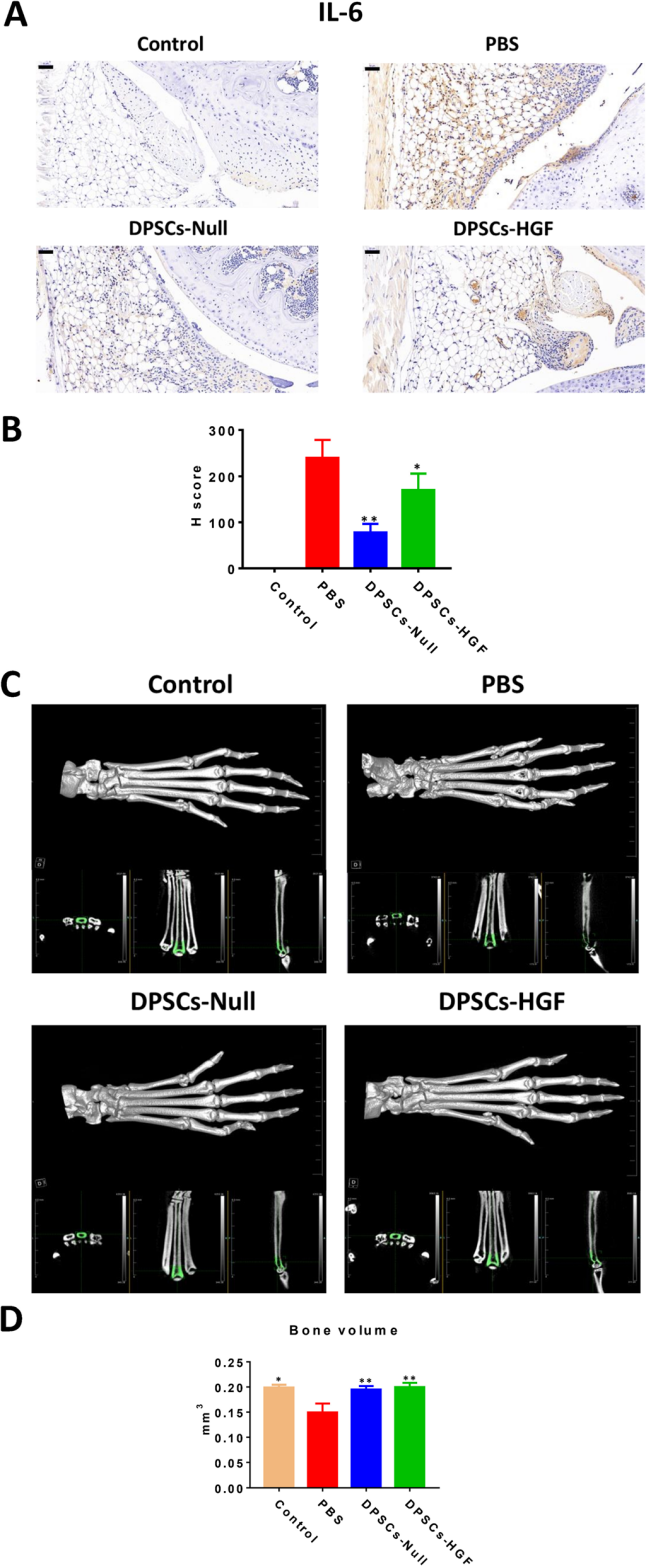
Immunohistologic staining and micro-CT results of CIA mice after different treatments. **a** Examples of IL-6 immunohistologic staining. Bar = 50 μm. The assessment was performed after the mice were sacrificed on day 49. **b** Statistic charts depict the H score of immunohistologic staining. * represents *p* < 0.05 (other groups vs. PBS treatment group). ** represent *p* < 0.01 (other groups vs. PBS treatment group). **c** Micro-CT results depict joint and bone radiologic changes in CIA mice after different treatments. Each picture contains the 3D reconstruction image, sagittal view, coronal view, and the transverse view of a specific claw. The green zone shows the area of interest measured for bone volume. The assessment was performed after the mice were sacrificed on day 49. **d** Statistic charts depict bone volume analysis by micro-CT. * represents *p* < 0.05 (other groups vs. PBS treatment group). ** represent *p* < 0.01 (other groups vs. PBS treatment group)

Since IL-6 in serum may not fully represent IL-6 levels in joints, we then performed IHC staining and calculated H scores to evaluate IL-6 in the joint (Fig. 4a, b). The results showed that synovial changes were related to the IL-6 level; the more IL-6 present, the heavier the synovitis develops (Fig. 4a). In addition, DPSCs extensively reduced IL-6 production in the joint, while overexpressing HGF in DPSCs reversed the inhibitory effects (Fig. 4a, b).

In addition, as a pathogenic factor in osteoclastogenesis, IL-6 has been reported to be responsible for local bone destruction in RA [24]. Therefore, to evaluate bone erosion, the right hind limbs of mice were collected and analyzed by the micro-CT system (Fig. 4c). Volumes of zones of interest (1 mm length in the proximal direction from the center of metatarsophalangeal joint, marked in green) were calculated (Fig. 4c, d). Compared with levels in the PBS treatment group, both the DPSCs-Null and DPSCs-HGF groups have reduced bone erosion in the CIA model (Fig. 4c). Intriguingly, the protection effect was not significantly different between the DPSCs-Null and DPSCs-HGF groups.

### HGF promotes IL-6 production, cell proliferation, and apoptosis resistance of FLSs

**Fig. 5:**
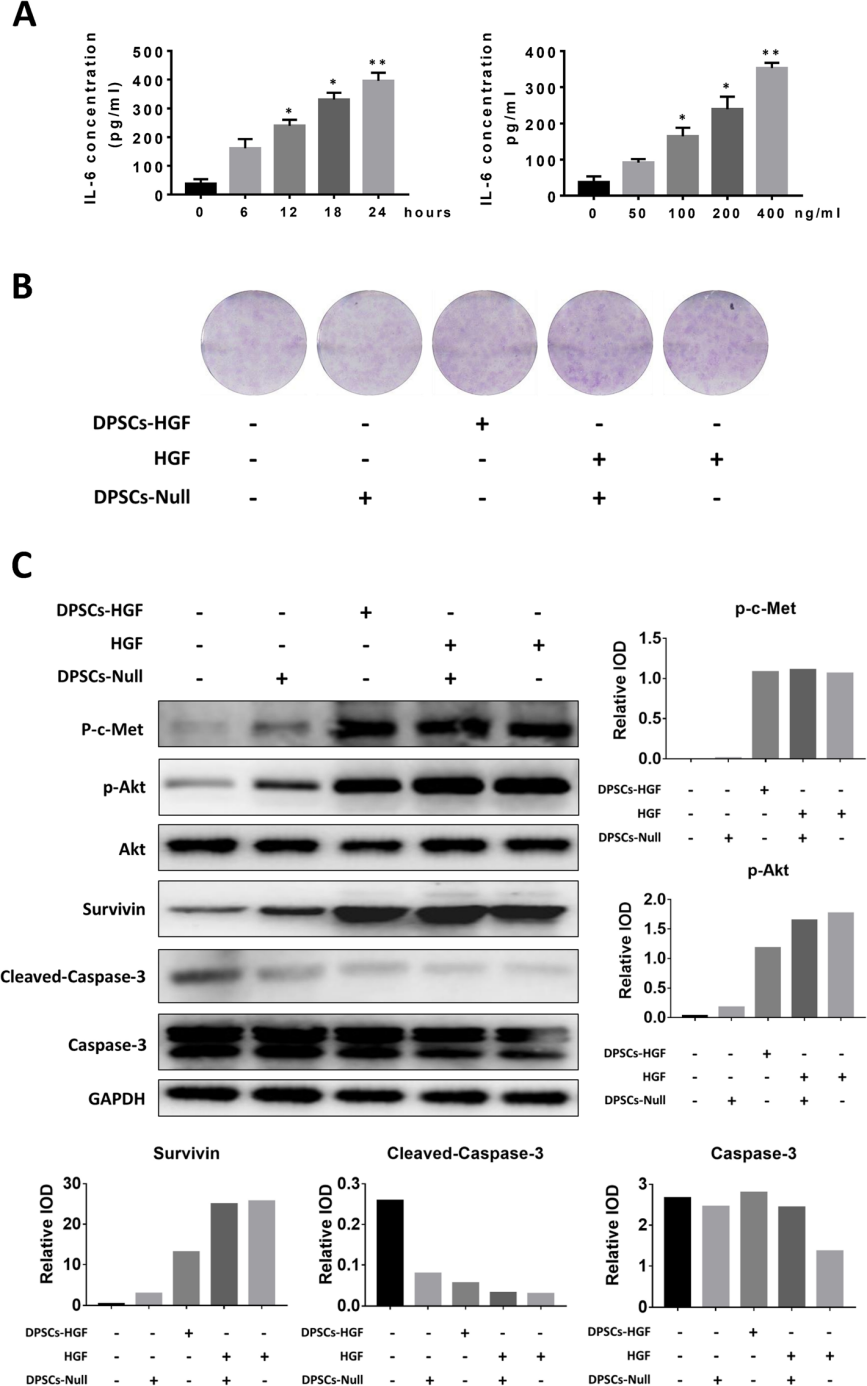
Biological effect of HGF on FLSs. **a** IL-6 production by FLSs was measured by enzyme-linked immunosorbent assay after treatment with HGF for a specific time or concentration. IL-6 level of FLS treated with 200 ng/ml HGF for different time (left). IL-6 level of FLS treated with different concentration of HGF for 12 h (right) * represents *p* < 0.05 (other groups vs. the first group). ** represent *p* < 0.01 (other groups vs. the first group). **b** Clonogenic analysis of FLSs after different treatments. “DPSC” represents synovial cells cultured in culture media containing DPSC culture supernatant (1:3 v/v). “HGF” represents synovial cells cultured in culture media containing HGF (100 ng/ml). “DPSCs-HGF” represents synovial cells cultured in culture media containing DPSCs-HGF culture supernatant plus HGF (1:3 v/v). After 21 days of culture, colonies were fixed and stained with Giemsa solution. **c** Western blot and IOD analysis results show the proteins expressed in FLSs after specific treatments. “DPSC” represents synovial cells cultured in culture media containing DPSC culture supernatant (1:3 v/v). “HGF” represents synovial cells cultured in culture media containing HGF (100 ng/ml). “DPSCs-HGF” means synovial cells cultured in culture media containing DPSCs-HGF culture supernatant plus HGF (1:3 v/v). After 12 h of treatment, cell lysate was analyzed by western blotting. For detection of cleaved Caspase-3, cells were treated with 1000 ng/ml TNF-α for 12 h before analysis. IODs were analyzed by Image Pro Plus 6. “Relative IOD” represents the IOD of a specific protein divided by the IOD of GAPDH

FLSs were considered a primary producer of local IL-6 in RA synovium; hence, we determined the effect of HGF on isolated FLSs from patients. After FLSs had been treated with HGF at different doses (0, 50, 100, 200, and 400 ng/ml) or for different times (0, 6, 12, 18, and 24 h), IL-6 levels in the culture media were measured. As shown in Fig. 5a, HGF stimulated FLSs to produce IL-6 in a time- and dose-dependent manner.

In addition, the clonogenic ability of FLSs was analyzed under different culture conditions. After culture for 21 days, images of hematoxylin staining showed that the clonogenic ability of FLSs following treatment with DPSCs-HGF, DPSC and HGF, or HGF alone was elevated (Fig. 5b). The results suggest that FLS proliferation is enhanced by HGF stimulation.

In addition, because cleaved Caspase-3 is an indicator of apoptosis, we then detected its level in cells pretreated with different conditions. The western blot results showed that cleaved Caspase-3 remarkably decreased following treatment with DPSCs-HGF, DPSC plus HGF, or HGF alone (Fig. 5c). The results indicated that FLSs were less prone to apoptosis after HGF treatment.

### HGF activates the Akt pathway to promote FLS pathogenesis

To investigate the underlying mechanism of HGF on FLSs, we evaluated the phosphorylation of c-Met (HGF receptor). As Fig. 5c shows, p-c-Met increased tremendously with the presence of HGF (either from overexpression or exogenous supplementation), which suggests that HGF could bind to c-Met and induce its activation.

As a receptor tyrosine kinase (RTK), c-Met can phosphorylate downstream pathways (such as the Akt pathway), causing certain biological activities. In this case, p-Akt was detected as an indicator of Akt pathway status. The results demonstrated that the activation of c-Met by HGF treatment caused the phosphorylation of the Akt pathway (Fig. 5c). In addition, we also detected Survivin, which is an inhibitor of apoptosis, an inducer of proliferation, and a potential downstream molecule of Akt. The results showed that HGF treatment (either from overexpression or exogenous supplementation) drastically induced Survivin expression.

To further confirm the influence of HGF, we utilized specific inhibitors (JNJ-38877605, LY294002, and YM-155) in the following experiments. JNJ-38877605 is an ATP-competitive inhibitor for HGF-stimulated c-Met phosphorylation. LY294002 remarkably inhibited PI3K/Akt activation, while YM-155 prevented Survivin expression.

**Fig. 6:**
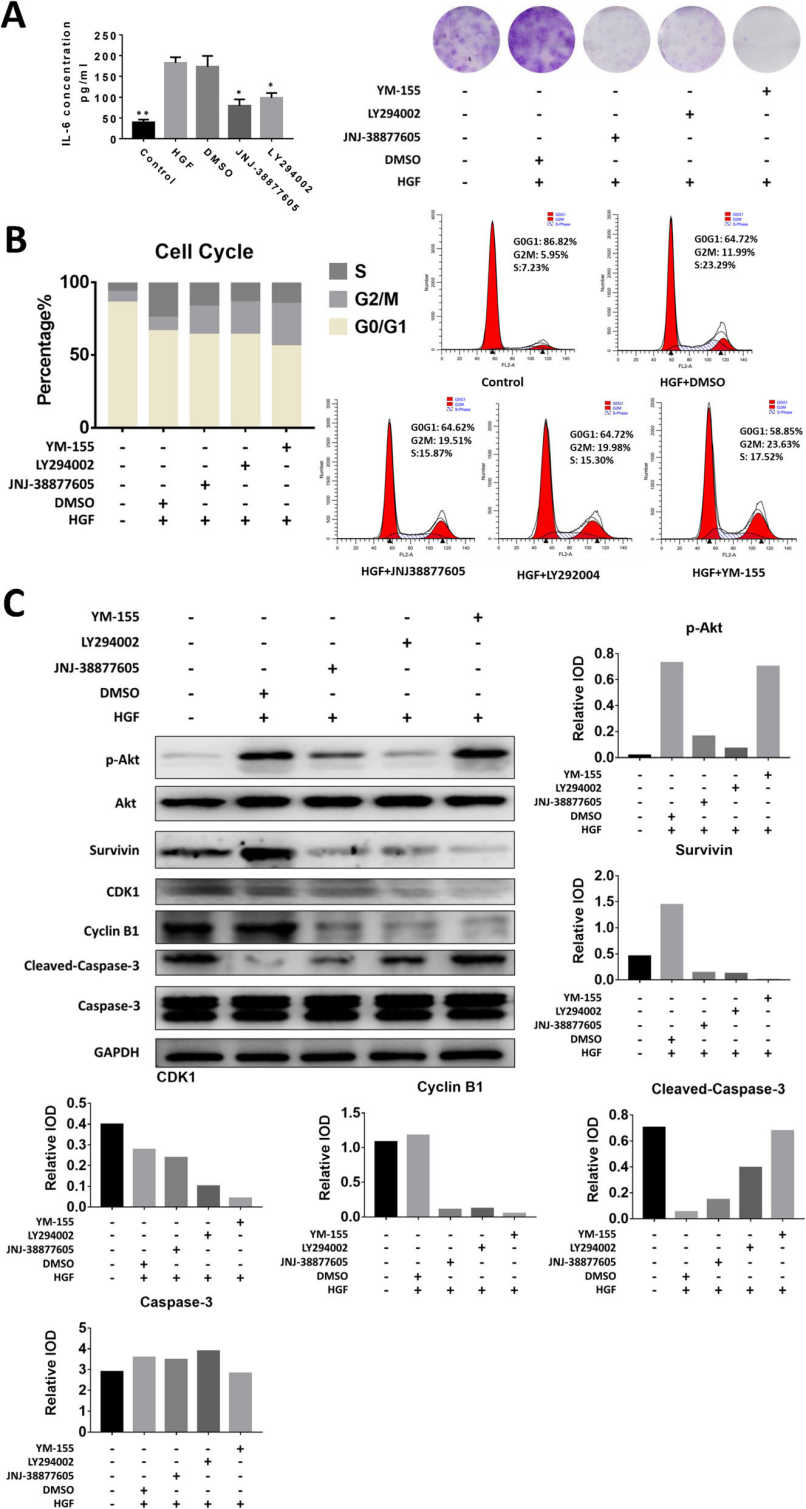
HGF/c-Met/Akt enhances the pathogenic effects of FLSs in RA. **a** IL-6 production and clonogenic ability of FLSs after treatment with HGF and/or DMSO (solvent control), JNJ-388777605 (c-Met pathway inhibitor), LY294002 (Akt pathway inhibitor), or YM-155 (survivin expression inhibitor). * represents *p* < 0.05 (other groups vs. HGF treatment group). ** represent *p* < 0.01 (other groups vs. HGF treatment group). **b** Cell cycle analysis after treatment with different inhibitors is shown. PI staining was utilized after treatment. **c** Western blot and IOD analysis results are shown of proteins expressed in FLSs after specific treatments. Cell lysates were analyzed by western blotting. IODs were analyzed by Image Pro Plus 6. “Relative IOD” represents the IOD of a specific protein divided by the IOD of GAPDH

The addition of JNJ-38877605 and LY294002 reduced IL-6 production (Fig. 6a, left). In addition, enhanced clonogenic abilities, as well as Survivin expression, of FLS by HGF stimulation were also reduced after inhibiting c-Met and Akt activation (Fig. 6a, right). Additionally, disrupting c-Met and Akt activation or Survivin expression caused G2/M cell cycle arrest in HGF-treated FLSs (Fig. 6b), which may be a possible reason for the ameliorated HGF-mediated clonogenic enhancement.

**Fig. 7:**
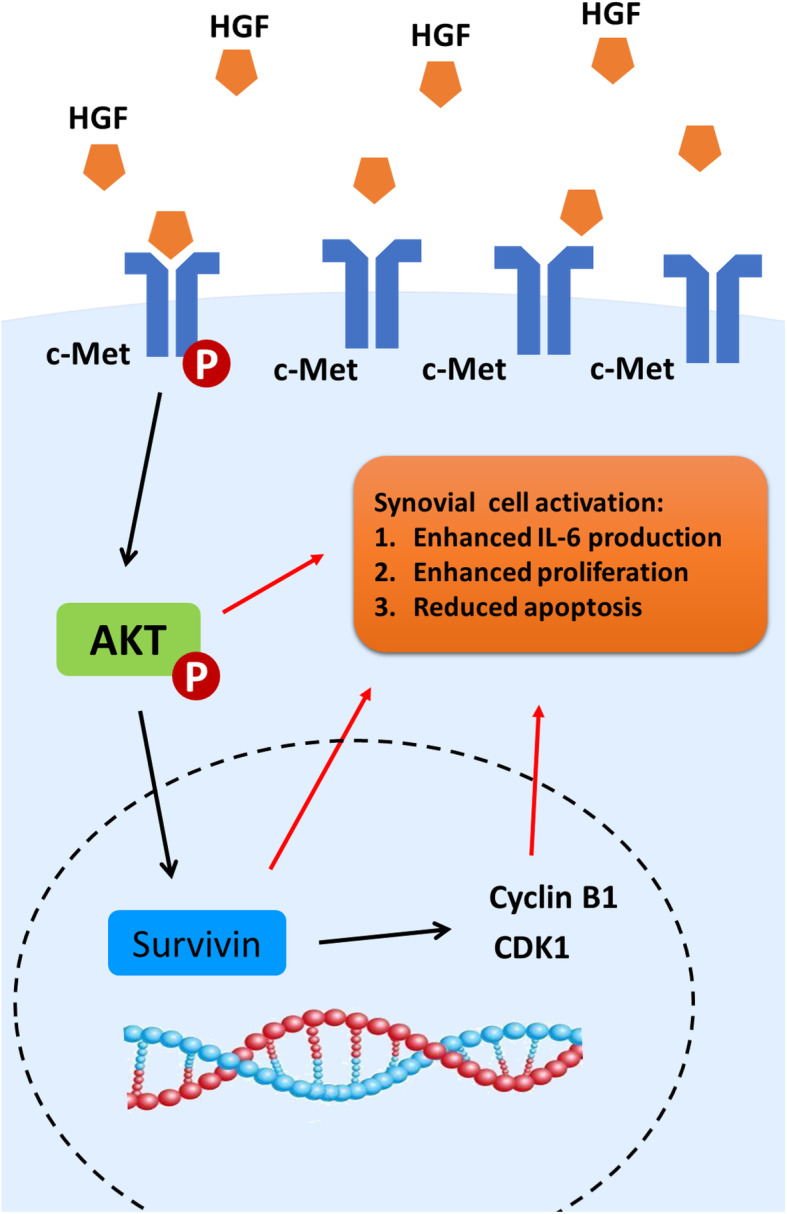
Schematic model of the mechanism underlying the effect of HGF on synovial cell activation. P, phosphorylation

After disrupting c-Met phosphorylation by JNJ-38877605 or Akt phosphorylation by LY294002, activation of the Akt pathway and expression of Survivin, CDK1, and Cyclin B1 decreased (Fig. 6c, lane 3) compared to that of cells treated with HGF and DMSO (Fig. 6c, lane 2), while cleaved Caspase-3 increased, indicating reduced proliferation and enhanced apoptosis. However, inhibition of Survivin expression could elevate cleaved Caspase-3 expression and reduce CDK1 and Cyclin B1 expression but not Akt activation, suggesting that Survivin is the downstream molecule of the Akt pathway but not the reverse (Fig. 6c, lane 5 and Fig. 7).

## Discussion

Recent preclinical studies have demonstrated the beneficial effects of MSCs in treating CIA mice [25]. Augello’s study revealed that because of the immunomodulatory effect, intraperitoneal injection of MSCs could prevent joint damage [26]. Other studies discovered that MSCs could inhibit T cell and B cell proliferation and differentiation, suppress dendritic cell maturation, and induce Tregs and anti-inflammatory macrophage polarization [27, 28]. However, as a type of MSC, DPSCs have been reported to possess stronger inhibitory abilities than those of stimulated T cells and higher regenerative potential than that of BMSCs [9, 10]. In addition, conditioned medium from human deciduous dental pulp stem cells has been reported to have a protective effect and could enhance anti-inflammatory M2 macrophage phenotype polarization in anti-collagen type II antibody-induced arthritis (another mouse model of RA) [29]. Because of these features, DPSCs have been considered promising stem cell sources for RA clinical utilization. In this study, we comprehensively evaluated the therapeutic effect of intravenous administration of DPSCs in treating CIA mice for the first time. We discovered that DPSC treatment could alleviate clinical manifestations and joint synovitis, reduce immune system activation and inflammatory factor production, and prevent bone loss and destruction in inflammatory arthritis.

HGF is a pleiotropic cytokine and a key player in mediating inflammatory and immune responses. HGF has been reported to influence multiple pathophysiological processes, such as immune cell migration and maturation, cytokine production, and T cell function, and to attenuate disease progression, including autoimmune neuroinflammation, autoimmune myocarditis, chronic graft-versus-host disease (GVHD), and lupus nephritis [30]. For RA, Okunishi et al. reported that HGF could suppress Ag-induced T cell priming by regulating dendritic cell functions and enhance the Th2-type immune response to inhibit the development of CIA [14]. Therefore, they believed that HGF is helpful in RA treatment. Intriguingly, different researchers claim that HGF is detrimental to RA. Shibasaki et al. showed that HGF and c-Met expression was elevated in RA synovium and that HGF stimulated MH7A cells (a human RA synovial cell line) to a tumor-like phenotype with elevated production of matrix metalloproteinase-3 (MMP-3) and vascular endothelial growth factor (VEGF) [31]. Additionally, blocking c-Met signaling was therapeutically helpful in inhibiting angiogenesis and cartilage and bone destruction [31] and inducing apoptosis of human rheumatoid arthritis FLSs [32]. In our study, we found that HGF has multifunctional effects on RA pathogenesis. In the early phase, HGF inhibited disease progression and demonstrated immunomodulatory effects. Treg cell elevation in the DPSCs-HGF treatment group occurred early but remained for a shorter duration than that of the DPSCs-Null group. However, in the late phase, HGF may be not protective but detrimental in RA progression. After HGF stimulation, serum and synovial IL-6 levels were elevated, and synovial cell proliferation and apoptosis resistance were enhanced, indicating that FLSs were activated after HGF stimulation.

Since synoviocytes are a key player in RA pathogenesis, to better understand the late detrimental effects of HGF in RA, we evaluated the role of the disease-related pathway on FLSs. After blocking c-Met or Akt pathway phosphorylation, the stimulatory effect of HGF was attenuated, indicating that these pathways participate in FLS activation after HGF stimulation. As the schematic model demonstrates (Fig. 7), HGF activates c-Met and the downstream Akt pathway, which promotes IL-6 production and induces Survivin, causing an accelerated FLS cell cycle and reduced apoptosis.

We believe that the multifunctional role HGF plays in RA causes the divergence of whether HGF is harmful or helpful in RA. In Okunishi’s study, HGF was given on day 0 and every 10 days [14], which meant that HGF was supplied before the CIA model had been established, indicating that HGF pretreatment before model induction may have a preventative effect on CIA due to its potent immunosuppressive effect. However, when HGF was administered after the CIA model had been established (consistent with our present study), the immunosuppressive effects of HGF on the immune system were impaired, while the pathological effects on synovitis were enhanced, causing activation of FLSs and deterioration of cartilage. This suggests that after CIA has developed to a certain level, HGF may be detrimental to disease progression.

IL-6 plays a crucial role in RA pathogenesis due to its proinflammatory properties [24]. IL-6 amplifies inflammatory cell infiltration, aggravates local inflammatory reactions, and accelerates synovitis and joint damage. FLSs have been reported to produce large amounts of IL-6 after stimulation by inflammatory cytokines such as IL-1, TNFα, and IL-17 [33]. However, no evidence has been put forward as to whether HGF induces IL-6 production in FLSs. In our study, we discovered that IL-6 was elevated locally after DPSCs-HGF treatment and that HGF stimulation promoted IL-6 production in a time- and dose-dependent manner. Interestingly, although IL-6 was considered a pathogenic factor for bone erosion, the elevated IL-6 after DPSCs-HGF treatment did not induce obvious bone destruction. This phenomenon might be a result of the fact that HGF could exert a protective effect. Kong et al. discovered that administration of DPSCs-HGF could promote the expression of bone formation-related genes and has stronger osteogenic differentiation capacities in ovariectomy-induced osteoporosis [11]. Likewise, we speculate that the protective effect of HGF neutralizes IL-6-induced bone destruction in the CIA model.

Overall, our study provides a comprehensive evaluation of the therapeutic effect of DPSCs in the CIA model for the first time. We found that DPSCs could control autoimmune status and protect against cartilage and bone destruction. In addition, we also discovered the multifunctional effect of HGF in RA. In the early phase, HGF inhibits RA progression by immunosuppressive effects, such as inducing Treg cells. In the late phase, HGF promotes synovitis by activating FLSs to produce pathogenic IL-6 and accelerating proliferation and inducing apoptosis resistance via phosphorylating the c-Met/Akt pathway.

## Conclusions

In this study, we provide a novel cell therapy by DPSC transfusion in RA, propose a new inducer of IL-6 production in RA, describe the dual role of HGF in RA pathogenesis, and provide a potential therapeutic target for RA treatment. Since the CIA model may not fully mimic RA pathogenesis in humans, further clinical research needs to be carried out to verify the above conclusions.

## Supplementary information

**Additional file 1.**

## Data Availability

All data generated and/or analyzed during this study are included in this published article. Data sharing is not applicable to this article as no datasets were generated or analyzed during the current study. However, the data that support the findings of this study are available from the corresponding author upon reasonable request.

## Abbreviations

RA: Rheumatoid arthritis
DPSCs: Dental pulp stem cells
HGF: Hepatocyte growth factor
CIA: Collagen-induced arthritis
FLSs: Fibroblast-like synoviocytes
DMARDS: Disease-modifying antirheumatic drugs
NSAIDs: Nonsteroidal anti-inflammatory drugs
MSCs: Mesenchymal stem cells
AdVs: Adenovirus vectors
CFA: Complete Freund’s adjuvant
CII: Type II collagen
IFA: Incomplete Freund’s adjuvant
Micro-CT: Microcomputed tomography
RTK: Receptor tyrosine kinase
GVHD: Chronic graft-versus-host disease
MMP-3: Matrix metalloproteinase-3
VEGF: Vascular endothelial growth factor
IOD: Integrated option density
