# Supplementary figures and images for: Pulp stem cells with hepatocyte growth factor overexpression exhibit dual effects in rheumatoid arthritis
Source: Stem Cell Res Ther. 2020 Jun 10;11:229. doi: 10.1186/s13287-020-01747-y (PMC7288412; doi:10.1186/s13287-020-01747-y)

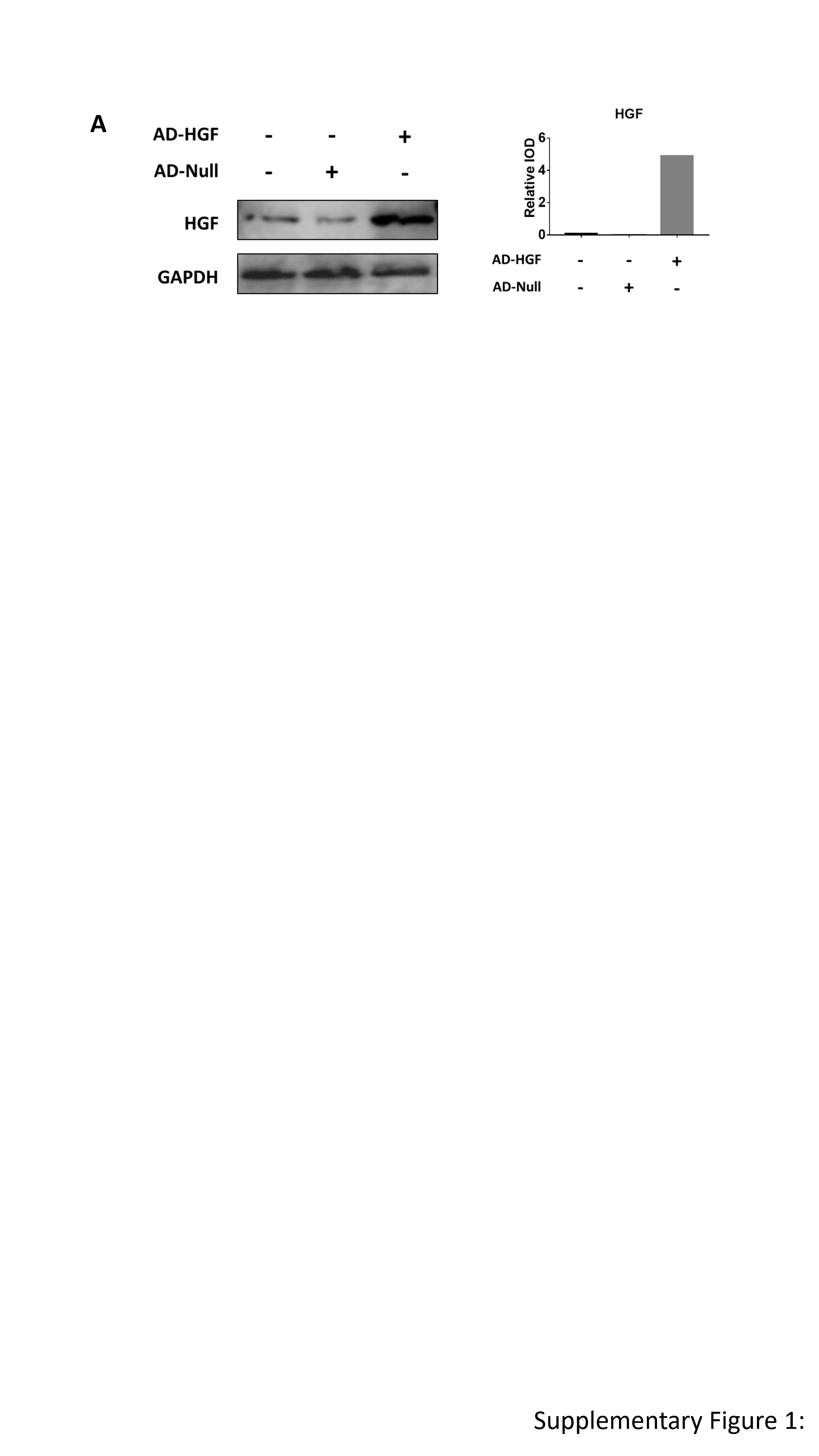

Supplement: Supplementary file 1 — Additional file 1. [file 13287_2020_1747_MOESM1_ESM.jpg]
